# *Staphylococcus epidermidis* activates keratinocyte cytokine expression and promotes skin inflammation through the production of phenol-soluble modulins

**DOI:** 10.1016/j.celrep.2023.113024

**Published:** 2023-08-22

**Authors:** Michael R. Williams, Michelle D. Bagood, Timothy J. Enroth, Zoie L. Bunch, Nina Jiang, Edward Liu, Samia Almoughrabie, Shadi Khalil, Fengwu Li, Samantha Brinton, Nadja B. Cech, Alexander R. Horswill, Richard L. Gallo

**Affiliations:** 1 Department of Dermatology, University of California, San Diego, San Diego, CA 92093, USA; 2 Department of Veterans Affairs Denver Health Care System, Denver, CO, USA; 3 Department of Immunology and Microbiology, University of Colorado Anschutz Medical Campus, Aurora, CO 80045, USA; 4 Department of Chemistry and Biochemistry, University of North Carolina Greensboro, Greensboro, NC 27402, USA; 5 Lead contact

## Abstract

*Staphylococcus epidermidis* is a common microbe on human skin and has beneficial functions in the skin microbiome. However, under conditions of allergic inflammation, the abundance of *S. epidermidis* increases, establishing potential danger to the epidermis. To understand how this commensal may injure the host, we investigate phenol-soluble modulin (PSM) peptides produced by *S. epidermidis* that are similar to peptides produced by *Staphylococcus aureus*. Synthetic *S. epidermidis* PSMs induce expression of host defense genes and are cytotoxic to human keratinocytes. Deletion mutants of *S. epidermidis* lacking these gene products support these observations and further show that PSMs require the action of the EcpA bacterial protease to induce inflammation when applied on mouse skin with an intact stratum corneum. The expression of PSMδ from *S. epidermidis* is also found to correlate with disease severity in patients with atopic dermatitis. These observations show how *S. epidermidis* PSMs can promote skin inflammation.

## In brief

Williams et al. show that the skin microbe *Staphylococcus epidermidis* can produce phenol-soluble modulins (PSMs) that will induce skin inflammation. These PSMs combine with cysteine proteases to promote skin disease.

## Graphical Abstract

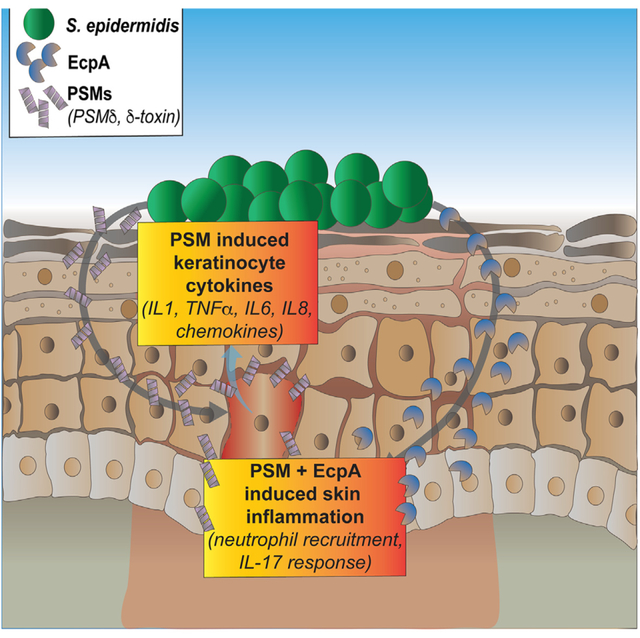

## INTRODUCTION

*Staphylococcus epidermidis* (SE) is the most commonly cultured bacteria from human skin and is described as having several potential beneficial effects including functions that improve wound healing, prevent tumor growth, recruit immune cells, promote skin barrier repair, and inhibit TLR3-dependent skin inflammation.^1–7^ Some strains of SE have also shown the ability to combat pathogenic microbial colonization by *S. aureus* through the production of lantibiotics and small accessory gene regulator (*agr*) quorum sensing autoinducing peptides.^2,4^ However, despite these potential beneficial effects of SE on the skin, this bacterial species can also result in infections when it penetrates the epidermal barrier and can contaminate implanted medical devices by forming antibiotic-resistant biofilms.^8–12^ More recently, it has been observed that SE may have non-infectious yet damaging effects when there is an increase in absolute and relative abundance of this organism in skin inflammatory disorders such as atopic dermatitis (AD) and Netherton’s syndrome^13,14^; a previously unappreciated complication of SE on the skin surface. Thus, under conditions of excess growth, SE may switch from a beneficial commensal to a harmful pathogen.

Given the potential beneficial and detrimental roles of SE in the human skin microbiome, a more detailed understanding of the mechanisms used by SE to interact with the host is needed to better understand the implications of its presence on human skin. Although overgrowth of SE is frequently observed on patients with AD, little is known about why a higher density of SE can induce skin inflammation. This contrasts with the closely related pathogen *Staphylococcus aureus* that is not often cultured from healthy human skin and a clear link between the presence of *S. aureus* and disease severity has been revealed by previous studies.^4,15,16^ Prior work has shown that an enzyme produced by SE known as EcpA can promote skin inflammation,^17^ which may result from many resident and non-resident immune cells responding to bacterial challenge and producing cytokines in response to EcpA. However, EcpA enzyme is not directly proinflammatory to keratinocytes and cannot adequately explain the action of SE on keratinocytes. Therefore, additional unknown products of SE may contribute to the toxic effects of these bacteria when present in high concentrations.

In this study, we hypothesized that the expression of toxins, known as phenol-soluble modulins (PSMs), by SE will damage keratinocytes and promote skin inflammation. This hypothesis was based on the observation that some SE PSMs are homologous to PSMs produced by *S. aureus* that have been shown to damage keratinocytes and promote disease.^4,15,16^ We found that human keratinocyte cytotoxicity and expression of host defense genes was induced by synthetic *S. epidermidis* PSMs. This evidence was further supported by genetic deletion of these gene products, which also revealed that PSMs required functional bacterial protease EcpA to induce inflammation in a skin exposure model *in vivo*. Finally, PSMδ expression from *S. epidermidis* correlated with disease severity in patients with AD. One explanation for our findings is that the induction of SE PSMδ by quorum sensing during high-density overgrowth can promote skin inflammation, a relationship that future studies can expand.

## RESULTS

### SE PSMs induce an inflammatory response in human keratinocytes

**Figure 1.**
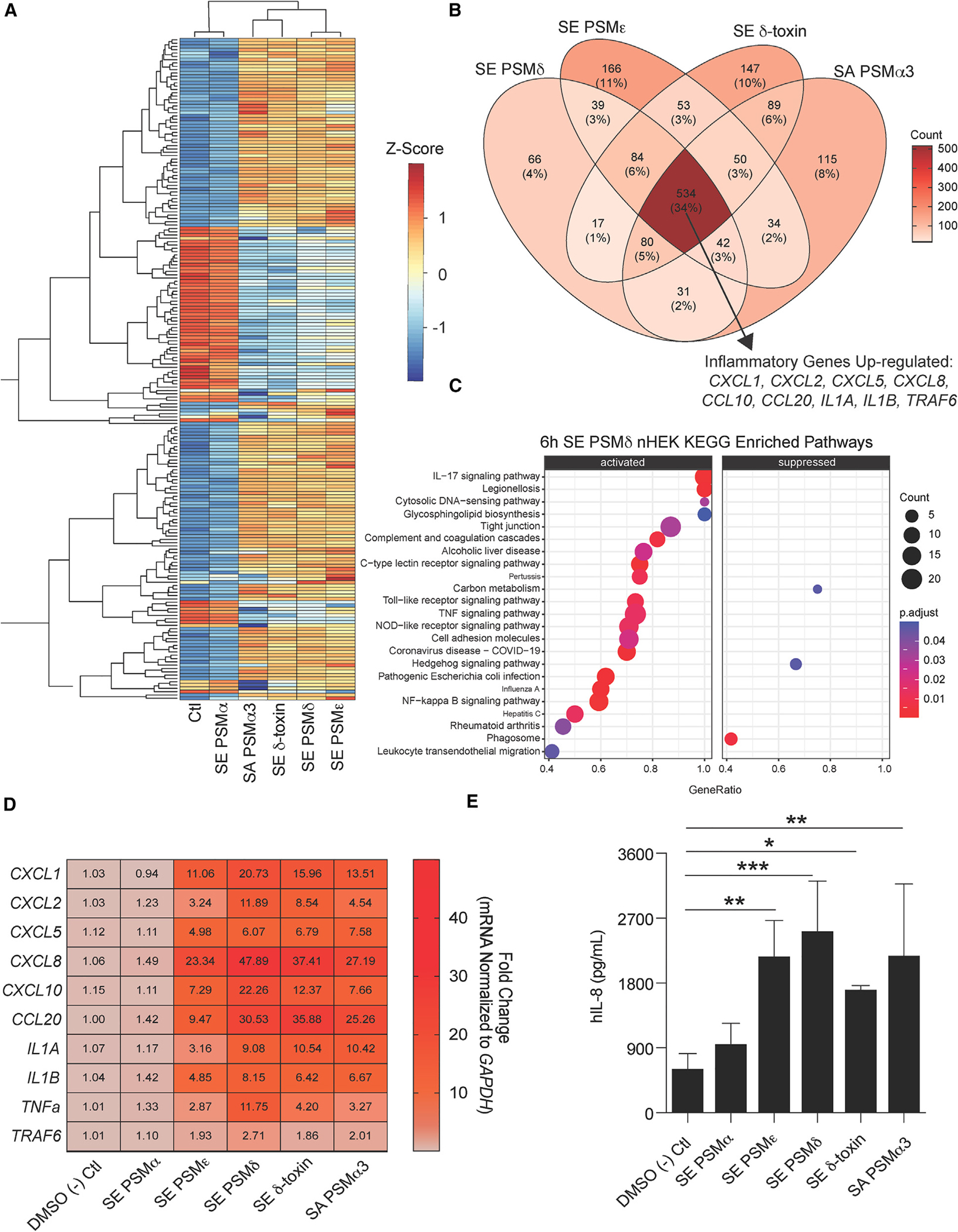
SE synthetic phenol-soluble modulins drive keratinocyte inflammation (A–C) RNA-seq data from primary human keratinocytes treated for 6 h at 10 μM with different *S. epidermidis* (SE) and *S. aureus* (SA) phenol-soluble modulins (PSMs). (A) Heatmap of top 250 variable genes. (B) Venn diagram of 2-fold up-regulated genes among PSMs differing from the control treatment with specific emphasis on up-regulated inflammatory genes shared among all PSMs. (C) Kyoto Encyclopedia of Genes and Genomes enriched pathway analysis of SE PSMδ (10 μM) treated keratinocytes. (D) qPCR results of keratinocytes treated for 6 h with SE and SA synthetic PSMs (10 μM) for gene significantly up-regulated in RNA-seq data above (n = 4). (E) ELISA analysis of human IL-8 cytokine (hIL-8) levels in conditioned medium from keratinocytes treated for 24 h with SE and SA synthetic PSMs (10 μM) (n = 4). Results are representative of at least two independent experiments. Mean ± SEM and a parametric unpaired one-way ANOVA analysis was used to determine statistical significance: *p < 0.05, **p < 0.01, ***p < 0.001, ****p < 0.0001. See also Figures S1A and S1B.

To determine the effect of SE PSMs on skin inflammation, primary human keratinocytes were treated for 6 h with 10 μM of synthetic SE PSM peptides. This included all the known α-helical peptides produced by SE, including PSMα, PSMδ, PSMε, and δ-toxin (*hld*). The homologous *S. aureus* PSMα3 peptide was used as a positive control since it is known to induce skin barrier damage and inflammation. Bulk RNA-sequencing (RNA-seq) analysis revealed that several SE PSMs including PSMδ, PSMε, and δ-toxin had markedly different gene expression profiles from the DMSO negative control treated keratinocytes and clustered closely with the known inflammatory *S. aureus* PSMα3 peptide. SE PSMα on the other hand displayed no change from the negative control treated cells (Figure 1A). Assessment of the 2-fold up-regulated genes from this bulk RNA-seq analysis also revealed that SE PSMs clustered with inflammatory *S. aureus* PSMα3 peptide and shared many genes in common (34%), including multiple inflammatory cytokines linked to the interleukin (IL)-17 signaling pathway such as CXCL1, CXCL2, CXCL5, CXCL8, CCL10, CCL20, IL1A, IL1B, and TRAF6 (Figures 1B and 1C). qPCR analysis of human keratinocytes treated for 6 h with 10 μM of these synthetic PSMs confirmed the up-regulation of these IL-17 signaling pathway genes with CXCL8 having the highest induction (Figure 1D). We then confirmed protein levels of the chemokine CXCL8 (hIL-8) were significantly increased by synthetic peptide treatment of keratinocytes (Figure 1E).

To provide insight into how the SE PSMs can activate keratinocyte inflammatory responses, we evaluated lactate dehydrogenase (LDH) release from human keratinocytes post treatment with PSMs and noted that this indicator of cytotoxicity peaked within 1 h of exposure to PSMδ (Figure S1A). This cytotoxic effect could be distinguished from the capacity of SE PSMδ to induce CXCL8 protein expression from keratinocytes as exposure to pertussis toxin, an inhibitor of G protein-coupled receptor (GPCR) signaling, did not affect LDH release at any time point (Figure S1A) but did significantly inhibit CXCL8 release at 6 and 24 h (Figure S1B). Overall these data show that multiple SE PSMs have the potential to drive production of inflammatory cytokines and chemokines and that SE PSMδ acts in part through the activation of GPCRs in human keratinocytes, not membrane permeabilization alone.

**Figure 2.**
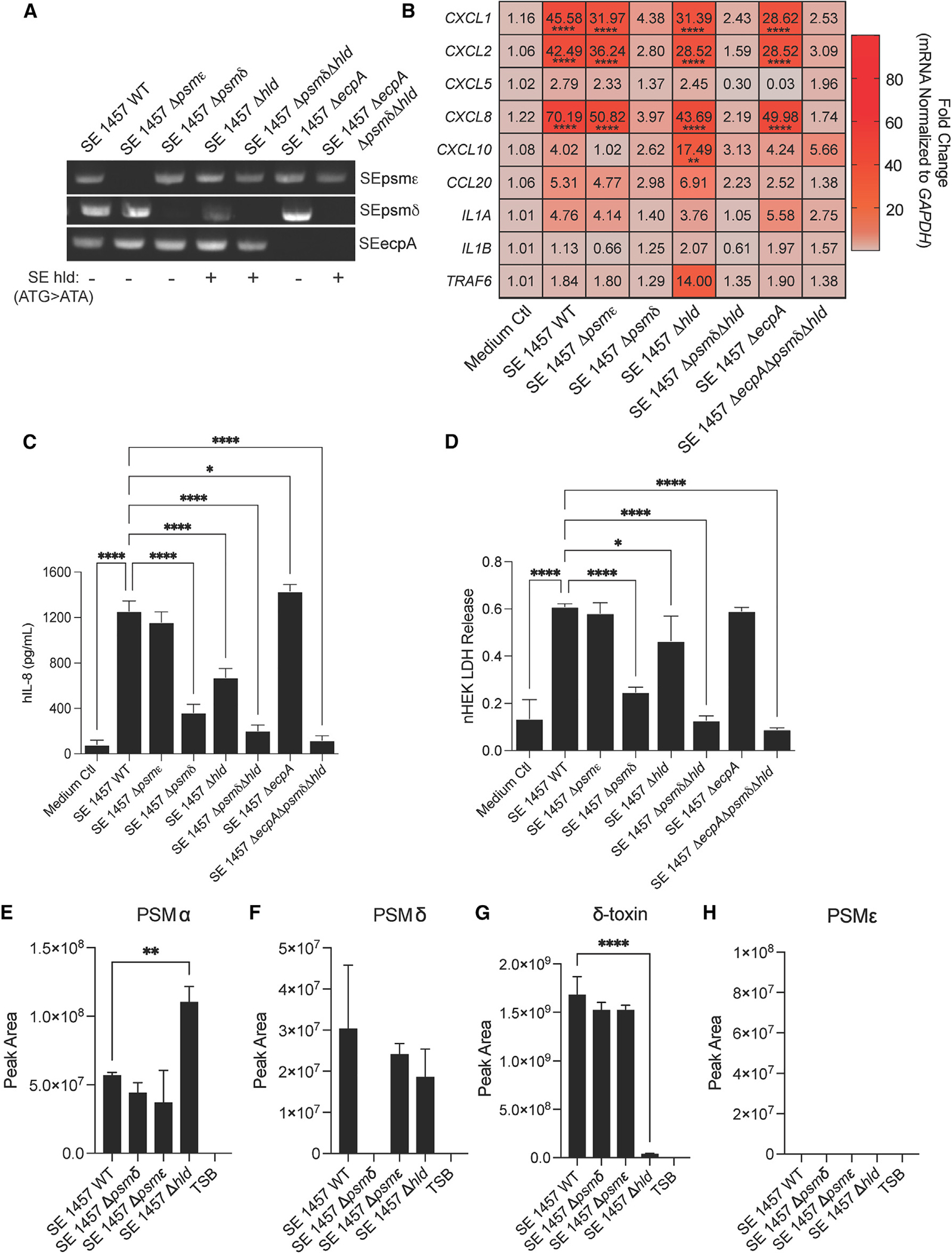
SE isogenic mutant strains reveal specific PSMs drive keratinocyte inflammation (A) PCR confirmation gel for specific SE knockout strains generated for this study. SE *hld* point mutation in start codon for gene confirmed through DNA sequencing and indicated by a +/−. For knockout strain growth curves, see also Figures S1C and S1D. (B) qPCR analysis of up-regulated inflammatory markers in keratinocytes treated for 3 h with 10% sterile-filtered conditioned medium of overnight growths of SE wild-type (WT) and specific knockout strains (n = 4). (C and D) (C) hIL-8 ELISA and (D) LDH release analysis from conditioned medium of keratinocytes treated for 24 h with 10% sterile-filtered conditioned medium of overnight growths of SE wild-type (WT) and specific knockout strains (n = 4). (E–H) Mass spectrometric validation of the presence or absence of PSMs in spent media for *S. epidermidis* 1457 WT and knockout strains. Results are averages of selected ion chromatogram peak areas for injections of biological triplicates of *S. epidermidis* spent media (TSB). The ions were detected in the quadropoly protonated form, [M+4H]4+. Results are representative of at least two independent experiments. Mean ± SEM and a parametric unpaired one-way ANOVA analysis was used to determine statistical significance: *p < 0.05, **p < 0.01, ***p < 0.001, ****p < 0.0001. See also Figure S2.

To further define which PSMs secreted from SE are responsible for inducing keratinocyte inflammation, a series of isogenic deletion strains were generated in SE using a previously published allelic replacement strategy.^18^ Single gene deletions were generated in the parent strain SE 1457 for several toxin genes including *psmδ* (Δ*psmδ*), *psm*ε (Δ*psm*ε), and *hld*, the gene for δ-toxin (Δ*hld*), as well as the cysteine protease gene *ecpA* (Δ*ecpA*). Different combinations of these knockout strains were also generated, including a double gene deletion (Δ*psm*δΔ*hld*) and a triple gene deletion (Δ*psm*δΔ*hld*Δ*ecpA*). All strains were confirmed to harbor the proper mutation by both PCR and DNA sequencing (Figure 2A), and growth of these strains was confirmed to be similar to each other (Figures S1C and S1D). To test the ability of each strain to modify primary human keratinocyte inflammation, 10% final volume of sterile-filtered supernatant from overnight culture of each strain was applied to keratinocytes for 24-h qPCR analysis, which revealed that inflammatory genes that were up-regulated by synthetic SE PSMs were also increased by the culture supernatant of the SE 1457 wild-type (WT) strain. The SE Δ*psmδ* knockout strain lost expression of these markers. Interestingly, deletion of the other two SE PSMs that could synthetically induce skin inflammation, Δ*psm*ε and Δ*hld*, as well as deletion of the cysteine protease EcpA (Δ*ecpA*), had a minimal effect on transcript expression (Figure 2B). Analysis of IL-8 protein expression and LDH release further revealed roles individually for SE *psmδ* and SE *hld* genes (Figures 2C and 2D). The double knockout for both genes Δ*psmδ*Δ*hld* prevented IL-8 secretion, while deletion of *psm*ε and *ecpA* had no effect (Figures 2C and 2D).

The absence of an effect on IL-8 protein expression (Figure 2C) for the SE Δ*psm*ε knockout strain was unexpected given that synthetic PSMε strongly induced IL-8 expression (Figure 1E). We hypothesized that these results might be explained by low production of PSMε by SE. To test this hypothesis, mass spectrometric analysis was conducted for spent media from the SE Δ*psm*ε, SE Δ*psmδ*, and SE Δ*hld* mutant strains, and the SE 1457 WT strain to measure levels of PSMα, PSMα, δ-toxin, and PSMε (Figures 2E–2H). While the PSMα level increased when the *hld* gene was deleted in SE 1457 (Figure 2E), we do not think it is responsible for the keratinocyte phenotype in the presence of SE Δ*hld* conditioned media (Figures 2C and 2D). We base this conclusion on the presence of *psm*ε, *psmδ,* and *ecpA* in the SE Δ*hld* knockout (Figure 2A) and the lack of keratinocyte phenotype with the PSMα synthetic peptide (Figure 1). PSMδ was detected in the WT strain (SE WT) and its identity was confirmed by matching fragmentation pattern with a synthetic SE PSMδ peptide (Figure S2A). As expected, PSMδ was not detected in the SE Δ*psmδ* knockout (Figure 2F). Similarly, and consistent with the data in Figure 2, SE δ-toxin was detected in the WT strain but not the knockout (Figure 2G) and its identity was confirmed by its fragmentation pattern with the standard (Figure S2B). A standard of synthetic SE PSMε peptide was detected by mass spectrometry (Figure S2C) but was not detected in spent media for WT SE 1457 or any of its mutants (Figure 2H). These results suggest that the absence of an effect observed for the SE Δ*psm*ε knockout (Figures 2C and 2D) is due to low production of PSMε by SE 1457. Overall, our data show that two specific SE PSMs drive inflammatory responses by keratinocytes *in vitro*, SE PSMδ (*psmδ*) and δ-toxin (*hld*), while minimal effects were seen after direct exposure of keratinocytes to SE culture supernatant after the deletion of genes for the other PSMs or the protease EcpA.

### SE cysteine protease EcpA drives early skin inflammation

**Figure 3.**
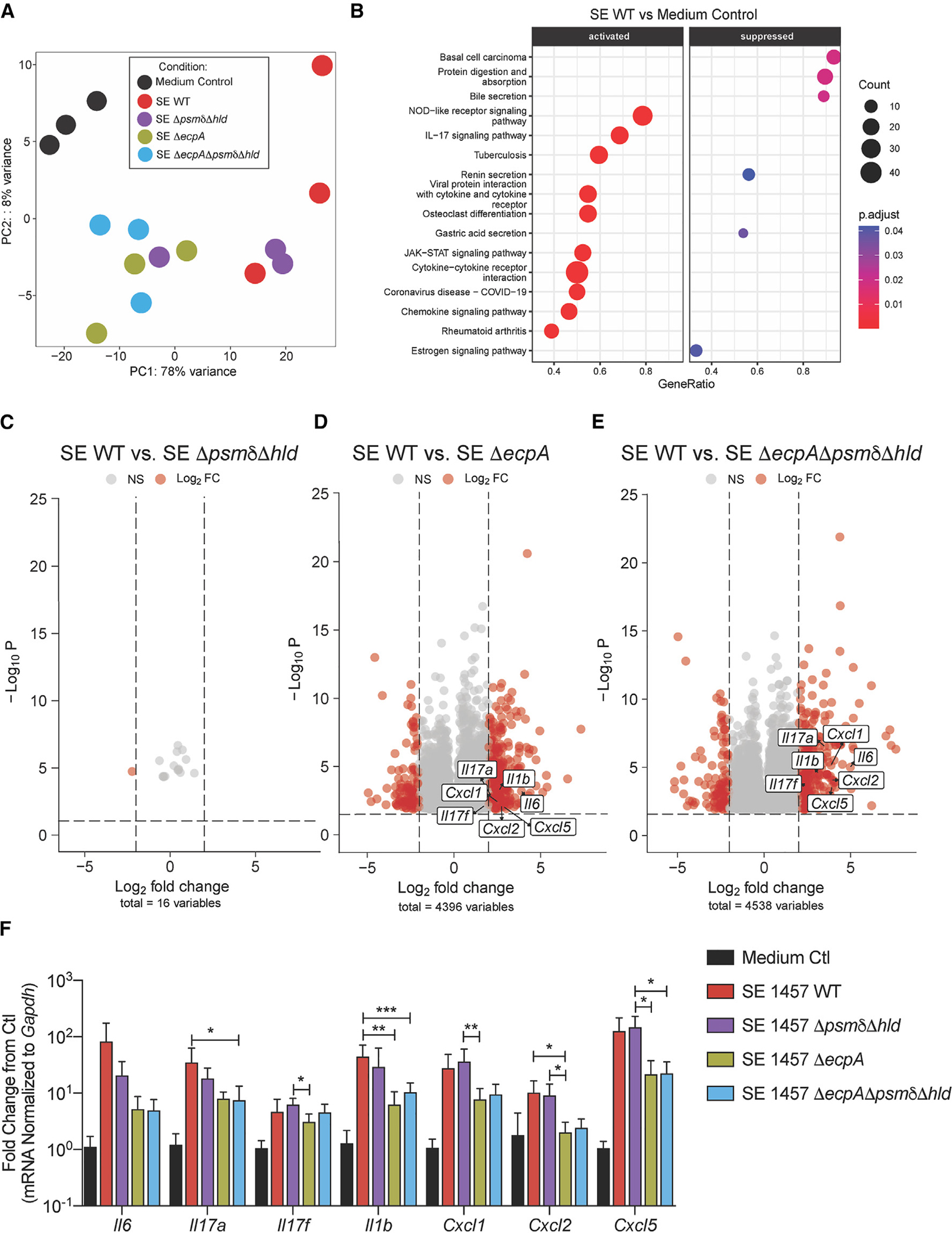
SE protease EcpA primarily drives early inflammation in epicutaneous mouse skin model (A) PCA analysis of bulk RNA-seq data from murine dorsal skin treated with epicutaneous application of 1 × 10^7^ CFU/cm^2^ of SE wild-type or mutant strains SE Δ*psmδ*Δ*hld*, SED*ecpA*, or SE Δ*psmδ*Δ*hl*dΔ*ecpA* for 24 h (n = 3 per group). (B) Kyoto Encyclopedia of Genes and Genomes enriched pathway analysis of SE WT versus control treated mouse skin at 24 h (C–E) Volcano plots of log2fold changed genes (red) between SE WT and various knockout (KO) strains. (F) qPCR analysis of up-regulated inflammatory genes in murine skin treated for 24 h with epicutaneous application of 1 × 10^7^ CFU/cm^2^ of SE wild-type or mutant strains SE Δ*psmδ*Δ*hld*, SE Δ*ecpA*, or SE Δ*psmδ*Δ*hld*Δ*ecpA* for 24 h (n = 6 per group). Results are representative of at least two independent experiments. Mean ± SEM and a non-parametric unpaired Kruskal-Wallis analysis was used to determine statistical significance: *p < 0.05, **p < 0.01, ***p < 0.001, ****p < 0.0001.

Having established that two SE PSMs, SE PSMδ (*psmδ*) and δ-toxin (*hld*), promote inflammatory cytokine responses from human keratinocytes *in vitro*, we next investigated how these PSMs affect mouse skin models of bacterial exposure, described in detail in the methods. Briefly, we removed hair by trimming and applying Nair, then allowed the skin barrier to recover for 48 h. Next, we epicutaneously applied SE at 1 × 10^7^ colony-forming units (CFU)/cm^2^ in 112.5 μL on a 1.5-cm^2^ piece of sterile 2 ply-gauze covered by Tegaderm and a band-aid to hold the microbial gauze in place for a short-term 24 h treatment on murine back skin to explore early transcriptional effects. Direct comparisons were made between SE 1457 WT and isogenic mutant strains including SE Δ*psm*δΔ*hld,* Δ*ecpA*, and the triple knockout strain (Δ*psmδ*Δ*hld*Δ*ecpA)* to account for effects of both PSMs and EcpA on the skin. These strains colonized the skin to a similar absolute level of live CFU (Figure S3A). At this early time point, disease severity trended downward with Δ*psmδ*Δ*hld* and Δ*ecpA,* but the most significant difference from the WT was observed with the combined triple deletion Δ*psmδ*Δ*hld*Δ*ecpA* (Figures S3B and S3C). Analysis of RNA-seq results from whole skin biopsies at 24 h was consistent with these visual assessments of inflammation and revealed transcriptional profiles at PC1 (representing 78% of the variance) from mice treated for 24 h with WT or SE Δ*psmδ*Δ*hld* were most distinct from medium only control mice (not treated with SE) (Figure 3A). In contrast, SE Δ*psmδ*Δ*hld*Δ*ecpA* and SE Δ*ecpA* were closer to medium only control mice and differed most along PC2, which represented only 8% of the variance. Notable increased expression of inflammatory genes in the IL-17 signaling pathway, including *Cxcl1, Cxcl2, Cxcl5, Il6, Il17a, Il17f,* and *Il1b,* were observed in mouse skin treated with the SE WT strain (Figure 3B). Assessment of the different SE knockout strains compared with the SE WT control revealed that SE *ecpA* deletion (Δ*ecpA)*, either alone or in combination with PSM gene deletion, altered host gene expression at 24 h (Figures 3C–3F).

### SE PSMs act after EcpA to amplify skin inflammation

**Figure 4.**
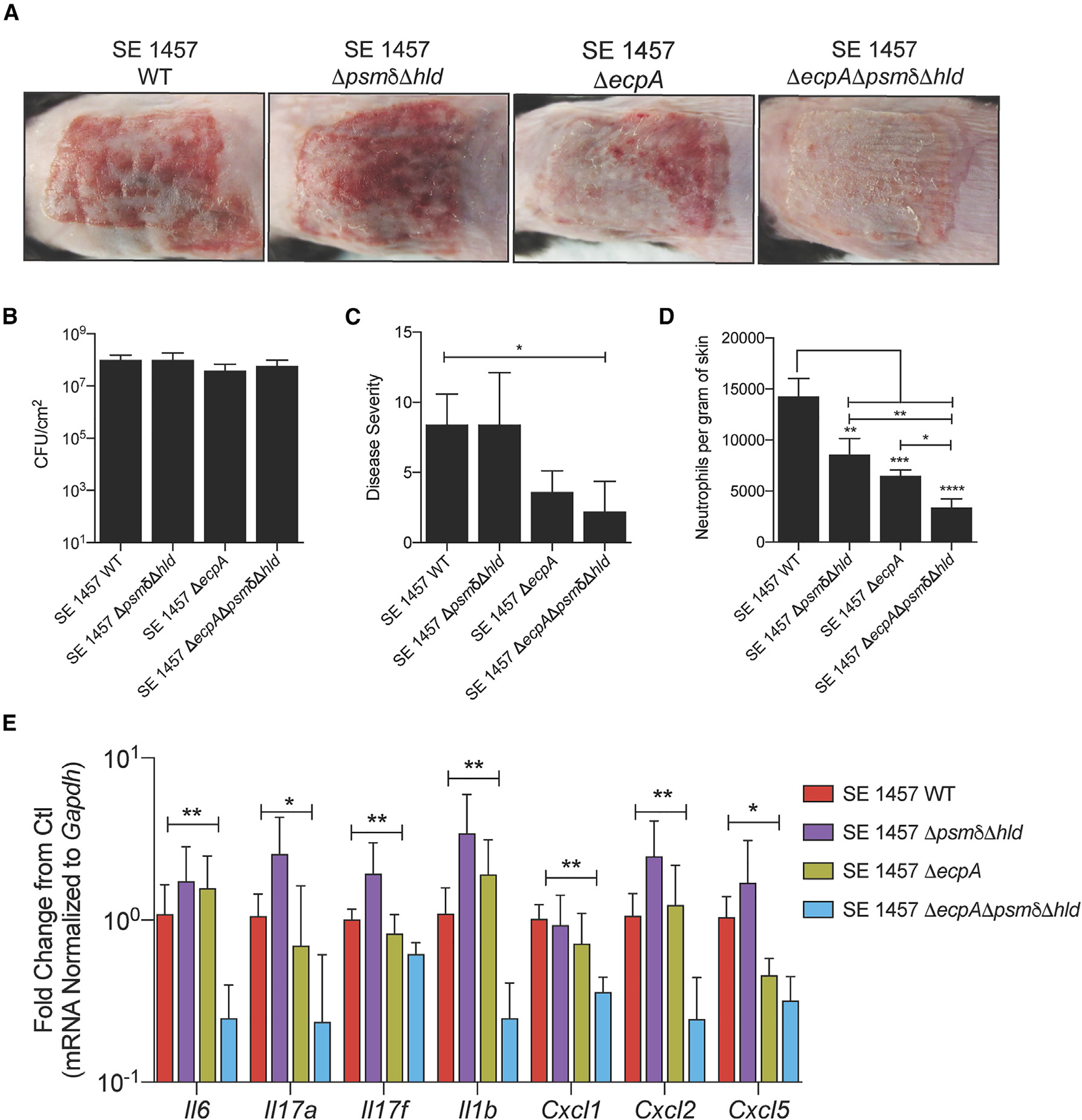
Both SE PSMs and EcpA promote skin inflammation in late inflammatory epicutaneous mouse model (A) Representative pictures of murine back skin after epicutaneous application of 1 × 10^7^ CFU/cm^2^ of SE wild-type (WT), SE Δ*psmδ*Δ*hld*, SE Δ*ecpA*, or SE Δ*psmδ*Δ*hld*Δ*ecpA* for 72 h (n = 5 per group). (B and C) CFU/cm^2^ of live bacteria and single-blinded assessment of skin disease severity following the 72-h application of epicutaneous bacteria. See also Figure S3. (D) Flow cytometric analysis of neutrophils (CD45^+^,CD11b^+^,Ly6G^+^) per gram of skin following the 72-h application of epicutaneous bacteria. Results represent mean ± SEM and a parametric unpaired one-way ANOVA analysis was used to determine statistical significance: *p < 0.05, **p < 0.01, ***p < 0.001, ****p < 0.0001. (E) qPCR analysis of up-regulated inflammatory genes in murine skin treated for 72 h with epicutaneous application of 1 × 10^7^ CFU/cm^2^ of SE wild-type or mutant strains SE Δ*psmδ*Δ*hld*, SE Δ*ecpA*, or SE Δ*psmδ*Δ*hld*Δ*ecpA* (n = 5 per group). Results are representative of at least two independent experiments. Mean ± SEM and a non-parametric unpaired Kruskal-Wallis analysis was used to determine statistical significance: *p < 0.05, **p < 0.01, ***p < 0.001, ****p < 0.0001.

To further test the effects of SE PSMs and EcpA protease after prolonged exposure to the skin, we utilized the *in vivo* model described briefly above and in full detail in the methods section and increased the exposure time of the skin to WT and knockout strains of SE to 72 h to better model AD. The SE WT strain induced robust skin inflammation at 72 h while the Δ*ecpA* and Δ*psmδ*Δ*hld*Δ*ecpA* mutants had decreased inflammation despite similar bacterial burden (Figures 4A–4C). However, in contrast to the minimal effects seen at 24 h (Figure S3), at 72 h the triple knockout strain showed less neutrophil recruitment to the skin and reduced expression of cytokines in the IL-17 signaling pathway compared with Δ*ecpA* (Figures 4D and 4E). These data suggest that both EcpA and PSMs (PSMδ and δ-toxin) contribute to the ability of SE to induce skin inflammation.

### Expression of SE *psmδ* correlates with disease in AD

**Figure 5.**
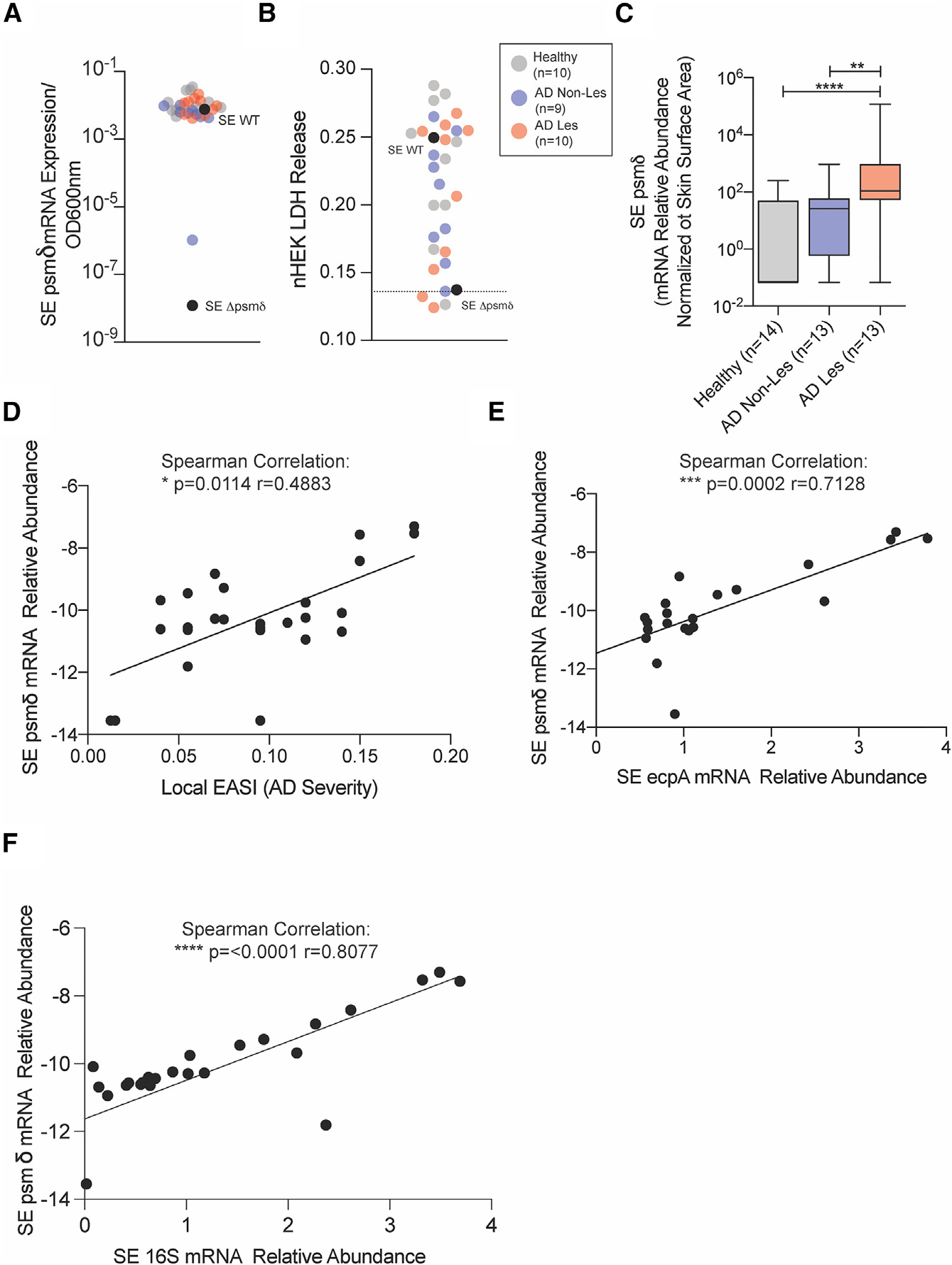
*Staphylococcus epidermidis* PSMs are produced in clinical isolates and elevated on diseased skin (A) SE *psmδ* transcript level expression compared with SE 1457 WT and SE 1457 Δ*psmδ* control strains across healthy (n = 10), atopic dermatitis non-lesional (AD Non-Les, n = 9), and atopic dermatitis lesional (AD Les, n = 10) clinical isolates. (B) Assessment of LDH release from keratinocytes treated for 24 h with the sterile-filtered conditioned medium from individual SE clinical isolates. (C) qPCR analysis of SE *psmδ* mRNA expression levels from skin swabs of healthy (n = 14), AD Non-Les (n = 13), and AD Les (n = 13) skin. (D–F) Spearman correlations between SE *psmδ* transcript levels and (D) local EASI disease severity scores, (E) SE *ecpA* transcript levels, and (F) SE 16s transcript levels on AD lesional skin swabs. Mean ± SEM and a non-parametric unpaired Kruskal-Wallis analysis was used to determine statistical significance: *p < 0.05, **p < 0.01, ***p < 0.001, ****p < 0.0001. See also Figure S4.

The increased absolute abundance of SE has been observed on AD lesional and non-lesional skin compared with normal skin and can reach levels similar to *S. aureus* (Figures S4A and S4B).^17^ To determine if such clinical isolates of SE express *psmδ*, we isolated RNA from overnight cultures of SE strains isolated from skin swabs obtained from the forearm of 10 healthy subjects and subjects with AD. qPCR analysis of RNA isolated from these strains determined that all clinical strains tested could express the *psmδ* gene when cultured similarly to SE 1457 WT (Figure 5A). Sterile-filtered conditioned medium from these samples were then applied to primary human keratinocyte cultures to evaluate if activity of these clinical isolates was like the laboratory isolate SE 1457 used in prior experiments. Analysis of LDH release revealed that most SE clinical isolates induced keratinocyte cytotoxicity (Figure 5B), suggesting similar cytotoxic effects of these isolates.

Next, qPCR analysis of SE *psmδ* transcript levels were measured directly from mRNA extracted from skin swabs. This analysis revealed that SE *psmδ* mRNA expression was significantly elevated on AD lesional skin compared with non-lesional and healthy skin samples (Figure 5C), as was SE *ecpA* (Figure S4). Furthermore, *psmδ* and *ecpA* expression levels on AD lesional skin correlated with disease severity as measured by Local EASI (Figure 5D, S4C, and S4D), and *psmδ* was correlated with *ecpA* (Figure 5E) and SE 16S expression levels (Figure 5F). This analysis of SE *psmδ* levels on diseased skin showed that not only is SE *psmδ* increased on diseased skin, but also it is associated with increased disease severity and co-expressed with *ecpA*.

## DISCUSSION

SE is a major component of the normal flora residing on human skin and has been primarily investigated in health care settings due to its important roles in opportunistic infections. Recent studies, including this one, have shown that, like the closely related organism *S. aureus*, SE increases in abundance on the skin of Th2 inflammatory disorders such as AD.^13,17^ This study further expands information for how SE can cause skin inflammation by demonstrating that the production of a group of toxins called PSMs induces keratinocyte cytokine expression and amplifies skin inflammation. The expression of PSMs was observed to directly damage primary human keratinocytes and to induce a transcriptomic response characteristic of staphylococcal infections of the skin. Further analysis in mouse models demonstrated that the effects of SE PSMs are enabled by the simultaneous expression of EcpA protease, thereby enabling PSMs to exert their proinflammatory effects through an intact epidermal barrier. Since both PSMs and EcpA are dependent on quorum sensing and regulated by the *agr* system,^19^ these observations may show how overgrowth of SE on the skin can cause skin disease, which is another potential mechanism by which SE can contribute to disease.

Our *in vitro* data in primary human keratinocytes demonstrated how multiple synthetic SE PSMs induced a pattern of inflammatory gene expression that was similar to that of *S. aureus* PSMα3, a previously defined skin toxin. This included up-regulation of IL-17 pathway specific cytokines and chemokines known to recruit immune cells to the skin including CXCLs and IL-1 cytokines.^15,16,20–22^ Interestingly, not all synthetic peptides shown to induce skin inflammation in keratinocytes could produce an effect when isogenic mutants to these PSMs were generated and their secreted products applied to cultured keratinocytes. Genetic deletion of SE *psm*ε did not influence keratinocyte cytokine transcript levels, protein production, or cell cytotoxicity when compared with the response to the parental SE 1457 strain. In contrast, deletion of genes for PSMδ or δ-toxin (*hld*) from SE 1457 greatly reduced the capacity of each isogenic mutant to stimulate an inflammatory response in keratinocytes. The lack of an effect from deletion of the gene for SE PSMε, despite the activity of the synthetic peptide, is likely due to the relatively low production of SE PSMε by this specific strain (SE 1457), as shown by mass spectrometry analysis, and may not be similar in other strains of SE. However, observations of synthetic peptides show that, like some PSMs produced by *S. aureus*, SE PSMε, PSMδ, PSMα, and δ-toxin could all be important epidermal toxins and can potentially drive skin inflammation. Although reagents are not currently available to measure protein abundance of these toxins on the skin of a large population of patients, or a wide variety of SE strains, this would be of interest to evaluate in the future.

Upon epicutaneous application of SE to mice, an interesting phenotype was revealed regarding the effect of SE PSMs on an intact skin barrier that may also inform how *S. aureus* acts in disease. We found that at early time points (24 h), the absence of SE PSMs (Δ*psmδ*Δ*hld*) had virtually no effect on inflammation or the expression of inflammatory cytokines in mouse skin. At this initial phase of skin injury by SE, the cysteine protease EcpA was necessary. However, SE PSMs did appear to have an important role at later time points (72 h), after the barrier was disrupted. At this stage, PSM expression influenced changes in visual signs of skin inflammation, immune cell recruitment, and transcriptional changes to IL-17 regulated immune signals. We hypothesize that unlike the human keratinocyte model where cells are directly in contact with PSMs, the stratum corneum barrier of intact skin prevented PSMs from penetrating without the aid of EcpA. The breakdown of the skin barrier by the proteolytic action of EcpA^14,17^ may have enabled PSMs to penetrate the skin at 72 h and further induce disease, possibly by acting directly to recruit PMNs as has been shown with *S. aureus* PSMs.^23^ Therefore, both toxins and protease expression by SE is important in driving inflammation of the skin.

*S. aureus* is considered a pathogen in AD due to a strong association with disease severity. However, at any one time, the presence of *S. aureus* is not detectable by culture methods from the skin of approximately 50% of patients with AD, thus prompting the question if other organisms on the skin of patients with AD may contribute to disease. SE is a prime candidate to act in the absence of *S. aureus* as it is most similar genetically from among the coagulase-negative staphylococcal species and SE is frequently also increased on the skin of AD.^13^ Our observations show that in addition to the increase in SE abundance there is also an increase in mRNA for PSMs. Expression is highest on lesional skin and the abundance of mRNA correlated with disease severity. Since both PSM gene and *ecpA* expression are under control of the *agr* quorum sensing system, these data may explain how an elevated density of SE on the skin can lead to production of these damaging toxins, a relationship that can be revealed by expanding this study. This may be a key element in understanding that why SE can exist on healthy skin without causing damage may be that the low density on normal skin will limit the expression of SE PSMs. In contrast, during disease, the increase in SE abundance enables activation of the *agr* system and increased expression of PSM genes as well as *ecpA*. Turning on the *agr* system may be sufficient to switch SE from a beneficial microbe to one that acts as a pathogen on the skin.

In summary, this study highlights the importance of SE and its PSMs in driving skin inflammation both *in vitro* and *in vivo* and solidifies the damaging effects of an overgrowth of SE on the skin. We provide evidence that SE PSMs can induce inflammation and may exacerbate disorders such as AD. Understanding the mechanisms of action behind both the beneficial and the damaging effects of skin microbes such as SE can improve therapeutic interventions that target the skin microbiome and skin inflammation.

### Limitations of the study

This study sought to better understand the mechanisms by which SE can damage human skin. The methods used included cultured primary human keratinocytes and mouse models. Although these systems have been often used to predict the response of human skin, neither completely reproduce the structure of human skin or differentiation state of keratinocytes in the epidermis. The influence of other microbes that are present on human skin have not been accounted for in these studies. Furthermore, technical difficulties prevented successful complementation of deletion mutants used in this study, thus raising the potential that the phenotypes observed in these experiments may not be solely the result of mutation at a single genetic locus. This limitation may be of particular biological relevance, as there is not full concordance between extracellular complementation studies with synthetic peptides and the phenotype of isogenic mutants, potentially highlighting the complexity of PSM regulation *in vivo*.

## STAR★METHODS

### RESOURCE AVAILABILITY

#### Lead contact

Further information and requests for resources and reagents should be directed to and will be fulfilled by the lead contact, Richard L. Gallo M.D., Ph.D. (rgallo@ucsd.edu).

#### Materials availability

There are restrictions to the availability of the *S. epidermidis* mutant strains produced for this paper due to biohazardous regulations. For distribution of materials, we require written explanation of the request and use (i.e., MTA).

#### Data and code availability

- Bulk RNA-seq data have been deposited at GEO and are publicly available as of the date of publication. Accession numbers are listed in the key resources table.
- This paper does not report original code.
- Any additional information required to reanalyze the data reported in this paper is available from the lead contact upon request.

### EXPERIMENTAL MODEL AND STUDY PARTICIPANT DETAILS

#### Human subjects

Experiments involving human subjects were done according to protocols approved by UCSD (University of California, San Diego) IRB (Project#140144). Written informed consent was obtained from all subjects. Swabs of surface microbiota from a 5 cm^2^ area of the antecubital fossa skin of both left and right arms were collected from 14 healthy subjects and 13 patients with AD as previously described.^17^ For subjects with AD, swabs were collected from both lesional and non-lesional skin.

#### Bacteria models

All bacteria used in this study are listed in the key resources table. All *Staphylococcus epidermidis* strains were grown overnight (18 h) to stationary phase in 3% TSB at 300 rpm in a 37°C incubator unless stated otherwise. All staphylococci indicated were grown approximately to an OD600nm reading of 10 or 3 × 10^9^ CFU/mL. For the treatment of bacterial supernatant on primary neonatal human epidermal keratinocytes, bacteria cultured overnight were pelleted (5 min, 4000 rpm, room temperature) and the supernatant was filter sterilized (0.22 μm) prior to addition to cells. For mouse experiments with sustained high exposure to live bacteria, bacterial CFU was approximated by OD600 nm before application to mouse back skin followed by confirmation of the actual CFU the following day.

#### Mouse model of epicutaneous bacterial exposure

All animal experiments were approved by the UCSD Institutional Animal Care and Use Committee (Protocol#S09074). Eight-week-old male C57BL/6 (The Jackson Laboratory) female mice were used for all experiments (n = 5 per condition), as specified in the figure legends. Mice were co-housed with n = 5 per cage for all experiments. Mouse hair was removed by trimming and applying Nair for 2 min followed by immediate removal with alcohol wipes. The skin barrier was allowed to recover from hair removal for 48 h before application of live bacteria. Staphylococcal strains were applied to the skin at 1 × 10^7^ CFU/cm^2^ in 3% TSB medium for 24 or 72 h at 112.5 μL volume on a 1.5-cm^2^ piece of sterile 2 ply-gauze. Tegaderm was applied on top of gauze along with a band-aid to hold the microbial gauze in place for the duration of the treatment. The severity of skin inflammation was single-blindly assessed from murine dorsal skin photographs and quantified using a total disease score as done previously.^31^

#### Primary normal human epidermal keratinocytes

Neonatal primary human epidermal keratinocytes (nHEKs) (Thermo Fisher Scientific) were cultured in EpiLife medium containing 60 μM CaCl_2_ (Thermo Fisher Scientific) supplemented with Human Keratinocyte Growth Supplement (Thermo Fisher Scientific) and antibiotic-antimycotic [penicillin (100 U/ml), streptomycin (100 U/ml), amphotericin B (250 ng/mL); Thermo Fisher Scientific] at 37°C, 5% CO_2_. For experiments, nHEKs were only used for experiments between passages 3–5 and grown to 70% confluency followed by differentiation in high-calcium EpiLife medium (2 mM CaCl_2_) for 48 h to recapitulate the upper layers of the epidermis *in vitro*. For bacterial supernatant treatments, differentiated NHEKs were treated with sterile-filtered bacterial supernatant at 10% by volume to EpiLife medium for up to 24 h. This volume was selected to ensure secreted product treatment while limiting cytotoxicity of bacteria culture media in keratinocyte media. Similarly, for synthetic PSM treatments, the peptides were added to the nHEKs for up to 24 h in a final volume of 0.1% dimethyl sulfoxide (DMSO) and 10 μM concentration.

### METHOD DETAILS

#### RNA isolation and quantitative real-time PCR

RNA was isolated using the PureLink RNA isolation kit according to manufacturer’s instructions (Thermo Fisher Scientific). For human keratinocytes, 250 μL of RNA lysis buffer was added directly to cells prior to addition of 250 μL of 70% EtOH and column based isolation of RNA. For mouse tissue, full thickness skin was subjected to bead beating in 750 μL of RNA lysis buffer (3 × 30sec with 5min on ice in between, 2.0mm zirconia bead). Tissue was then centrifuged (10min, 13,000RPM, 4°C), followed by adding of 350 μL of clear lysate to 70% EtOH and column based isolation of RNA. For bacterial RNA SE psmδ transcript levels, 500μL of overnight bacteria growth was added to 1mL of RNA Protect Bacteria Reagent (Qiagen) for 10 min at RT and pelleted (13,000RPM, 10′, RT). Pellet was resuspended in 700uL of RNA lysis buffer along with bead beating (3 × 30sec with 5min on ice in between, 0.1mm zirconia bead), centrifugation (13,000, 10′, RT), and transfer of 350 μL of clear lysate to 70% EtOH and column based isolation of RNA. After RNA isolation, samples were quantified with a Nanodrop (ThermoFisher Scientific), and 750ng of human or mouse RNA was reverse-transcribed using the iScript cDNA synthesis kit (Bio-Rad). qPCR reactions were run on a CFX96 Real-Time Detection System (Bio-Rad). For both human and mouse cDNA, 2x SYBR Green qPCR Master Mix was used along with specific primers as indicated in key resources table.

Microbial DNA was extracted from skin swabs by using the PureLink Microbiome DNA Purification Kit (Thermo Fisher Scientific). The absolute abundance of *S. aureus* and SE gDNA in the microbial DNA elution was determined by qPCR as previously described.^4,15,16^ Briefly, qPCR was performed with iTaq Universal SYBR Green Supermix (Bio-Rad) by using SE (*sodA*) and *S. aureus* (*femA*) specific primers. To determine the relative CFUs of the specific DNA, a standard curve was generated with gDNA extracted from known CFUs of the *S. aureus* strain 113 and SE strain ATCC12228, respectively. The specificity of all primer pairs was confirmed by melting curve analysis and comparison with standard curves.

#### Enzyme-linked Immunosorbent Assay (ELISA) analysis

Supernatant from cell cultures was collected prior to RNA isolation and stored at −80°C until use. ELISA analysis was performed using a Human IL-8/CXCL8 DuoSet ELISA (R&D Systems) kit following the manufacturer’s instruction.

#### Flow Cytometry

Murine dorsal skin was stored 24 h in Tissue Storage Solution (Miltenyi Biotech) and digested using RPMI medium 5% Fetal Bovine Serum (FBS) with Antimycotic/Antibiotic containing Liberase TL (1.67 Wunsch unites/mL) and DNAse (500 μg/mL) rotating at 37°C for 2h followed by filter separation (100→70→30 μm). After a 5′ application at RT of red blood cell lysis buffer, single cells were resuspended in FACS buffer (DPBS containing 1% BSA) followed by surface staining for viable (Ghost Fixable Viability Dye; Tonbo) neutrophils (CD45^+^ (clone 30-F11; Biolegend), CD11b^+^ (clone M1/70; Biolegend), and Ly6G^+^ (clone 1A8; Biolegend)) positive cells. Cell acquisition was performed on a Bio-RAD ZE5 flow cytometer and data analyzed using FlowJo software (Treestar).

#### Synthetic S. epidermidis PSM preparation

All synthetic PSMs were produced by LifeTein. Peptides were produced at 95% purity with N-terminal formylation. PSM peptides were pre-aliquoted to 500mg and resuspended in DMSO to a stock concentration of 10 mM prior to further dilution in tissue culture growth medium for experiments. The peptide sequences were generated as below.

1. SE PSMα: fMADVIAKIVEIVKGLIDQFTQK.
2. SE PSMδ: fMSIVSTIIEVVKTIVDIVKKFKK.
3. SE PSMε: fMFIINLVKKVISFIKGLFGNNENE.
4. SE δ-toxin: fMAADIISTIGDLVKWIIDTVNKFKK.
5. *S. aureus* PSMα3: fMEFVAKLFKFFKDLLGKFLGNN.

#### S. epidermidis isogenic deletion by allelic replacement

A previously established allelic replacement procedure in *Staphylococcus epidermidis* (SE)^18^ was used to create isogenic *psmα, psmδ*, *psm*ε, *hld*, *ecpA*, and different combinations of mutants in a frequently used clinical isolate of SE strain 1457. Approximately 1 kb upstream and downstream regions flanking the genes of interest were amplified from SE 1457 genomic DNA using PCR oligonucleotide pairs, with SacII digestion sites at edges of primers directly upstream and downstream of the gene of interest to excise. Construction of the *hld* mutant (to prevent production of δ-toxin peptide without disrupting *agr* RNAIII activity) was constructed through introduction of a start codon mutation (ATG to ATA) with pJB38 as well. All primers used for generation of these knockout strains as listed in key resources table.

#### Mass spectrometry

Ultra-performance liquid chromatography-mass spectrometry (UPLC-MS) data were obtained using a Q Exactive Plus quadrupole-Orbitrap mass spectrometer (ThermoFisher Scientific) with a heated electrospray ionization source coupled to an Acquity ultra-performance liquid chromatography (UPLC) system (Waters). Spent media samples were injected onto an Acquity UPLC BEH C18 column (1.7 μm, 2.1 × 50 mm, Waters) at a flow rate of 0.3 mL/min and an injection volume of 5 μL. A binary solvent gradient of water (H_2_O) with 0.1% formic acid (A) and acetonitrile (CH_3_CN) with 0.1% formic acid (B) was used. The solvent gradient initiated with a 1.5-min isocratic hold at 20% B and was followed by a linear increase to 60% B over 5 min. The gradient was held isocratic from 6.5 min to 7.0 min and then increased to 100% B at 8.0 min. The column was washed at 100% B for 1 min and then returned to the starting conditions to allow for re-equilibration for 1.0 min prior to the next injection. The first 1.5 min of eluent was diverted to waste.

UPLC-MS data were collected in positive ionization mode over a scan range of *m/z* 300–2000, with a resolving power of 35,000. The instrument parameters were: capillary temperature, 256 C, spray voltage, 3.00 kV, sheath gas, 48 arbitrary units; auxiliary gas, 11 arbitrary units; spare gas, 2 arbitrary units; and probe heater temperature, 350°C. UPLC-MS/MS data were acquired over a full scan range of *m/z* 300–2000 using all ion fragmentation (AIF) with higher-energy collisional dissociation (HCD) at stepped normalized collision energies of 20, 25, and 27. Synthetic PSM standards were subjected to the same UPLC-MS analyses as the spent media. To confirm identity, the accurate mass, retention time, and fragmentation patterns of the standards were compared to the putative PSM ions observed in the spent media.

#### RNA-seq

mRNA was extracted with the PureLink RNA isolation kit (Thermofisher Scientific) from either neonatal human epidermal keratinocytes or murine dorsal skin followed by quality control and library preparation at the University of California San Diego Integrated Genomics Core (IGM) Facility. mRNA quality was assessed on an Agilent 2100 Bioanalyzer to identify samples with RIN ≥7 and an Illumina stranded mRNA prep kit was used. A Novaseq 6000 S4 platform was used yielding an average of 25 million stranded 100-bp paired-end reads per sample.

Read quality was checked using Multiqc.^32^ Reads were aligned using either the human reference genome GRCh38.p13 or mouse reference genome GRCm39 (Gencode) using STAR v. 2.7.10a.^30^ For human mRNA isolated from neonatal human epidermal keratinocytes, fold change differences were determined using pooled mRNA samples (n = 1) and the EdgeR v. 3.38.1 package^26–28^ with a default dispersion value while murine sample differential expression was determined from biological triplicates using the De-Seq2 package v. 1.36.0,^29^ both using R studio.^33^ For murine skin differentially expressed gene (DEG) analysis, cutoffs of log2fold change >2 with an adjust p < 0.05 were used. Gene set enrichment analysis (GSEA) was performed on significant DEGs between subject groups using the ClusterProfiler package.^34,35^

The accession number for the bulk RNA-seq datasets generated in the course of this project have been deposited at the National Center for Biotechnology Information Gene Expression Omnibus (GEO): GSE209653 and GSE210046 respectively. Any further details regarding these datasets will be made available upon request.

### QUANTIFICATION AND STATISTICAL ANALYSIS

Student’s t tests and One-way and Two-way ANOVAs (parametric and non-parametric) tests were used for statistical analysis as indicated in the figure legends. All statistical analysis was performed using GraphPad Prism Version 8.0 (GraphPad, La Jolla, CA). All data is presented as mean ± standard error of the mean (SEM) and a p value ≤0.05 considered significant.

## Supplementary Material

1

## Figures and Tables

**KEY RESOURCES TABLE:** 

REAGENT or RESOURCE	SOURCE	IDENTIFIER

Antibodies		

Mouse CD45 Antibody	Cell Signaling	Cat# 70257S; RRID:AB_2237886
Mouse CAMP Antibody	Nizet et al., 2001^24^	N/A
Mouse Neutrophil Antibody	Abcam	Cat# ab53457; RRID:AB_881409
Mouse CD45 Antibody (Clone:30-F11) FITC	Biolegend	Cat#103107; RRID:AB_312972
Mouse Ly6G Antibody (Clone:1A8) BV711	Biolegend	Cat#127643; RRID:AB_2564383
Mouse CD11b Antibody (Clone:M1/70) PE-Cy7	Biolegend	Cat#101215; RRID:AB_312793
Ghost Dye Violet 510 (Live/Dead Fixable Stain)	Tonbo	Cat#13-0870-T500

Bacterial and virus strains		

*Staphylococcus epidermidis 1457 WT(B1)*	Gallo (UCSD)	This Study
*Staphylococcus epidermidis 1457 Δpsmδ* (1A–1)	Gallo (UCSD)	This Study
*Staphylococcus epidermidis 1457 Δpsmε* (1B–1)	Gallo (UCSD)	This Study
*Staphylococcus epidermidis 1457 Δhld* (1C-1)	Gallo (UCSD)	This Study
*Staphylococcus epidermidis 1457 Δpsmδ Δhld* (D5-1)	Gallo (UCSD)	This Study
*Staphylococcus epidermidis 1457 ΔecpA* (1C-1(1))	Gallo (UCSD)	This Study
*Staphylococcus epidermidis 1457 Δpsmδ Δhld ΔecpA* (1D-2(1))	Gallo (UCSD)	This Study
*E. coli* Chemically Competent One Shot Top 10	Invitrogen	Cat#C404010
*E. coli* (DC10B)	Monk and Foster^18^	N/A

Chemicals, peptides, and recombinant proteins		

2-mercaptoethanol	Sigma-Aldrich	Cat# M6250
Antibiotic-Antimycotic (100×)	Gibco	Cat# 15240062
Bacto Agar	BD Biosciences	Cat# 214010
Calcium chloride dihydrate	Sigma-Aldrich	Cat# 22,350-6
Defined Trypsin Inhibitor (DTI)	Gibco	Cat# R-007-100
DPBS	Gibco	Cat# 14190-144
Human keratinocyte growth supplement (HKGS)	Gibco	Cat# S0015
EpiLife complete medium, with 60 M calcium	Gibco	Cat# MEPI500CA
RPMI medium	Gibco	Cat#11875093
Liberase TL	Roche	Cat# 5401020001
DNAse	Sigma	Cat#D5025
Fetal Bovine Serum	Corning	Cat#35010CV
Ethyl alcohol, Pure	Sigma-Aldrich	Cat# E7023
Formalin	Azer Scientific	Cat# PFNBF-20
Lysing Matrix B	MP Biomedical	Cat# 116911050-CF
Lysing Matrix D	MP Biomedical	Cat# 116913050-CF
Molecular biology grade TE buffer	Invitrogen	Cat# AM9849
RNAlater Stabilization Solution	Invitrogen	Cat# AM7021
Tryptic soy Broth (TSB)	Sigma-Aldrich	Cat# T8907-1KG
Trypsin/EDTA solution	Gibco	Cat# R-001-100
UltraPure distilled water	Invitrogen	Cat# 10977-015
Q5 Hot Start High Fidelity Polymerase Master Mix (2×)	NEB	Cat#M0494L
Ultrapure Agarose	Thermofisher Scientific	Cat# 16500500
1kb Plus DNA Ladder	NEB	Cat#N3200L
Sybr Safe DNA Gel Stain	Thermofisher Scientific	Cat#S33102
SYBR Green qPCR Master Mix (2×)	Biomiga	Cat# QP1311-02
Prolong Antifade Gold	Thermofisher Scientific	Cat# P36934
*S. epidermidis* PSMd, fMSIVSTIIEVVKTIVDIVKKFKK	Lifetein	N/A
S. epidermidis PSMα, fMADVIAKIVEIVKGLIDQFTQK	Lifetein	N/A
*S. epidermidis* PSMe, fMFIINLVKKVISFIKGLFGNNENE	Lifetein	N/A
*S. epidermidis* d-toxin (hld), fMAADMSTIGDLVKWMDTVNKFKK	Lifetein	N/A
Optima LC/MS Water	Fisher Scientific	CAS 7732-18-5
Optima LC/MS Acetonitrile	Fisher Scientific	CAS 75-05-8
Optima LC/MS Formic Acid	Fisher Scientific	CAS 64-18-6
Optima LC/M Methanol	Fisher Scientific	CAS 67-56-1

Critical commercial assays		

iSCRIPT cDNA synthesis Kit	BIO-RAD	Cat#1708891
PureLink RNA Mini Kit	Invitrogen	Cat# 12183025
PureLink Microbiome DNA Purification Kit	Invitrogen	Cat# A29790
Human IL-8/CXCL8 DuoSet ELISA	R&D Systems	Cat# DY208

Deposited data		

Human keratinocyte *S. epidermidis* PSM treatment RNA-seq (bulk)	This Study	GEO: GSE209653
Murine *S. epidermidis* PSM treatment RNA-seq (bulk)	This Study	GEO: GSE210046

Experimental models: Cell lines		

Human Epidermal Keratinocytes, neonatal (HEKn)	Gibco	Cat# C0015C

Experimental models: Organisms/strains		

Mouse: C57BL/6J	Jackson Laboratory	Strain: 000664

Oligonucleotides		

*S. epidermidis psmδ 1kb Upstream Forward (KpnI)*	5′-GTCCGGTACCCACAAATAATGTTGCACCCC-3′	This Study
*S. epidermidis psmδ 1kb Upstream Reverse (SacII)*	5′-GTTCCCGCGGGTTCATGACCTCCTTTCAAAAGG-3′	This Study
*S. epidermidis psmδ 1kb Down Forward (SalI)*	5′- GTCCGTCGACCGCTGAGAGTAATCATTAATTTGC-3′	This Study
*S. epidermidis psmδ 1kb Down Reverse (SacII)*	5′- GTCACCGCGGTTCTACATGGGCCTGG-3′	This Study
*S. epidermidis psmδ 1kb Internal Forward*	5′-AGCAAGAGTGTCAATGGTTAC-3′	This Study
*S. epidermidis psmδ 1kb Internal Reverse*	5′-CTAAGAAAGCGAGCCAAC-3′	This Study
*S. epidermidis psmε 1kb Upstream Forward (KpnI)*	5′-GTCCGGTACCGATTTATATCCATCTTCAACGATTGC-3′	This Study
*S. epidermidis psmε 1kb Upstream Reverse (SacII)*	5′-GTTCCCGCGG*GTGACTCACCTCCTATGTATTTG*-3′	This Study
*S. epidermidis psmε 1kb Down Forward (SacII)*	5′-GTCACCGCGGTAATAATTAAACTATTCTCTACTCCGCC-3′	This Study
*S. epidermidis psmε 1kb Down Reverse (SalI)*	5′-GTCCGTCGACGAGAATTATACACCCTAGACCTG-3′	This Study
*S. epidermidis psmε Internal Forward*	5′-CTACATGTTAAAAAGTTAGGGAAAG-3′	This Study
*S. epidermidis psmε Internal Reverse*	5′-GATAACGTTGTAGAAATACTTTCAC-3′	This Study
*S. epidermidis hld 1kb Upstream Forward (KpnI)*	5′- GTCCGGTACCGCAGCCGGTGCAAAAGATATTAC-3′	This Study
*S. epidermidis hld 1kb Upstream Reverse (ATG→ATT)*	5′- GATATCTGCTGCTATTATAACTTCACTCCTTTCG–3′	This Study
*S. epidermidis hld 1kb Down Forward (ATG→ATT)*	5′- CGAAAGGAGTGAAGTTATAATAGCAGCAGATATC–3′	This Study
*S. epidermidis hld 1kb Down Reverse (SalI)*	5′- CAGGGTCGACCATCTCGTGCCAATGTTACATGAGCCAGAC-3′	This Study
*S. epidermidis hld Internal Forward*	5′- CTCCTCAAGTGTCATTATAC-3′	This Study
*S. epidermidis hld Internal Reverse*	5′- GCATAGTTAAAGCCGTG-3′	This Study
*S. epidermidis ecpA 1kb Upstream Forward (EcoRI)*	5′-GTCCGAATTCCATTACACATGATGAAGAAGC-3′	This Study
*S. epidermidis ecpA 1kb Upstream Reverse (SacII)*	5′-GTTCCCGCGGTTCATTTCGTGTACTTTGATATTC-3′	This Study
*S. epidermidis ecpA 1kb Down Forward (SacII)*	5′-GTCACCGCGGTATAGAAAGGTGTGCTTATG-3′	This Study
*S. epidermidis ecpA 1kb Down Reverse (SalI)*	5′-GTCCGTCGACGTTAGTAGCATTACCAGTG-3′	This Study
*S. epidermidis ecpA Internal Forward*	5′-CAAAAGGATATTTTGCACTTAC-3′	This Study
*S. epidermidis ecpA Internal Reverse*	5′-GTGAAGTTTCTACTTCTACAG-3′	This Study
Murine *Gapdh* primers	Mm.PT.39a.1 (Integrated DNA Technology)	N/A
Murine *Il6* primers	Mm.PT.58.10005566 (Integrated DNA Technology)	N/A
Murine *Il17a* primers	Mm.PT.58.6531092 (Integrated DNA Technology)	N/A
Murine *Il17f* primers	Mm.PT.58.9739903 (Integrated DNA Technology)	N/A
Murine *Cxcl1* primers	Mm.PT.58.42076891 (Integrated DNA Technology)	N/A
Murine *Cxcl2* primers	Mm.PT.58.10456839 (Integrated DNA Technology)	N/A
Murine *Cxcl5* primers	Mm.PT.58.29518961.g (Integrated DNA Technology)	N/A
Murine *Cxcl10* primers	Mm.PT.58.43575827 (Integrated DNA Technology)	N/A
Murine *1L1b* primers	Mm.PT.58.41616450 (Integrated DNA Technology)	N/A
Human *GAPDH* primers	Hs.PT.39a.22214836 (Integrated DNA Technology)	N/A
Human *IL6* primers	Hs.PT.58.40226675 (Integrated DNA Technology)	N/A
Human *CXCL1* primers	Hs.PT.58.39039397 (Integrated DNA Technology)	N/A
Human *CXCL2* primers	Forward: 5′-CTGCTCCTGCTCCTGGTG-3′Reverse: 5′-AGGGTCTGCAAGCACTGG-3′	Sanzari et al., 2009
Human *CXCL5* primers	Hs.PT.58.41058007.g (Integrated DNA Technology)	N/A
Human *CXCL8* primers	Hs.PT.39a.22214836 (Integrated DNA Technology)	N/A
Human *CXCL10* primers	Hs.PT.58.3790956.g (Integrated DNA Technology)	N/A
Human *CCL20* primers	Hs.PT.58.19600309 (Integrated DNA Technology)	N/A
Human *IL1A* primers	Hs.PT.58.40913627 (Integrated DNA Technology)	N/A
Human *IL1B* primers	Hs.PT.58.1518186 (Integrated DNA Technology)	N/A
Human *TNFA* primers	Hs.PT.58.45380900 (Integrated DNA Technology)	N/A
Human *TRAF6* primers	Hs.PT.58.4313477 (Integrated DNA Technology)	N/A
*S. epidermidis* psmδ F (qPCR)	5′-ATGGCAGCAGATATC-3′	This Study
*S. epidermidis* psmδ R (qPCR)	5′-GAATTTATTAACTGTATCGATAATC -3′	This Study

Recombinant DNA		

pJB38 plasmid	Bose et al., 2013^25^	N/A
pJB38-SEΔpsmδ	This Study	N/A
pJB38-SEΔpsmε	This Study	N/A
pJB38-SEΔhld	This Study	N/A
pJB38-SEΔecpA	This Study	N/A

Software and Algorithms		

GraphPad Prism (version 8)	GraphPad Software	https://www.graphpad.com/
EdgeR	Robinson et al.^26^; McCarthy et al.^27^; Chen et al.^28^	https://bioconductor.org/packages/release/bioc/html/edgeR.html
Deseq2	Love et al.^29^	https://bioconductor.org/packages/release/bioc/vignettes/DESeq2/inst/doc/DESeq2.html
STAR (version 2.7.3a)	Dobin et al.^30^	https://github.com/alexdobin/STAR/releases
R (version 3.4.1)	R Core team, 2017	https://cran.r-project.org/bin/windows/base/old/3.4.1/

## Highlights

- *S. epidermidis* phenol-soluble modulins promote cytokine expression by keratinocytes
- *S. epidermidis* PSMs with EcpA induce murine Type 17 skin inflammation
- *S. epidermidis* PSM levels are elevated in human atopic dermatitis

## References

[1] Byrd AL, Belkaid Y, and Segre JA (2018). The human skin microbiome. Nat. Rev. Microbiol. 16, 143–155. 10.1038/nrmicro.2017.157. 29332945

[2] Cogen AL, Yamasaki K, Sanchez KM, Dorschner RA, Lai Y, MacLeod DT, Torpey JW, Otto M, Nizet V, Kim JE, and Gallo RL (2010). Selective antimicrobial action is provided by phenol-soluble modulins derived from Staphylococcus epidermidis, a normal resident of the skin. J. Invest. Dermatol. 130, 192–200. 10.1038/jid.2009.243. 19710683 PMC2796468

[3] Nakatsuji T, Chen TH, Butcher AM, Trzoss LL, Nam SJ, Shirakawa KT, Zhou W, Oh J, Otto M, Fenical W, and Gallo RL (2018). A commensal strain of Staphylococcus epidermidis protects against skin neoplasia. Sci. Adv. 4, eaao4502. 10.1126/sciadv.aao4502. 29507878 PMC5834004

[4] Williams MR, Costa SK, Zaramela LS, Khalil S, Todd DA, Winter HL, Sanford JA, O’Neill AM, Liggins MC, Nakatsuji T, (2019). Quorum sensing between bacterial species on the skin protects against epidermal injury in atopic dermatitis. Sci. Transl. Med. 11, eaat8329. 10.1126/scitranslmed.aat8329. 31043573 PMC7106486

[5] Naik S, Bouladoux N, Linehan JL, Han SJ, Harrison OJ, Wilhelm C, Conlan S, Himmelfarb S, Byrd AL, Deming C, (2015). Commensal-dendritic-cell interaction specifies a unique protective skin immune signature. Nature 520, 104–108. 10.1038/nature14052. 25539086 PMC4667810

[6] Zheng Y, Hunt RL, Villaruz AE, Fisher EL, Liu R, Liu Q, Cheung GYC, Li M, and Otto M (2022). Commensal Staphylococcus epidermidis contributes to skin barrier homeostasis by generating protective ceramides. Cell Host Microbe 30, 301–313.e9, e309. 10.1016/j.chom.2022.01.004. 35123653 PMC8917079

[7] Lai Y, Di Nardo A, Nakatsuji T, Leichtle A, Yang Y, Cogen AL, Wu ZR, Hooper LV, Schmidt RR, von Aulock S, (2009). Commensal bacteria regulate Toll-like receptor 3-dependent inflammation after skin injury. Nat. Med. 15, 1377–1382. 10.1038/nm.2062. 19966777 PMC2880863

[8] Dong Y, Speer CP, and Glaser K (2018). Beyond sepsis: Staphylococcus epidermidis is an underestimated but significant contributor to neonatal morbidity. Virulence 9, 621–633. 10.1080/21505594.2017.1419117. 29405832 PMC5955464

[9] Kretschmer D, Nikola N, Dürr M, Otto M, and Peschel A (2012). The virulence regulator Agr controls the staphylococcal capacity to activate human neutrophils via the formyl peptide receptor 2. J. Innate Immun. 4, 201–212. 10.1159/000332142. 22067547 PMC3388272

[10] Le KY, Park MD, and Otto M (2018). Immune Evasion Mechanisms of Staphylococcus epidermidis Biofilm Infection. Front. Microbiol. 9, 359. 10.3389/fmicb.2018.00359. 29541068 PMC5835508

[11] Otto M (2009). Staphylococcus epidermidis–the ‘accidental’ pathogen. Nat. Rev. Microbiol. 7, 555–567. 10.1038/nrmicro2182. 19609257 PMC2807625

[12] Uçkay I., Pittet D., Vaudaux P., Sax H., Lew D., and Waldvogel F. (2009). Foreign body infections due to Staphylococcus epidermidis. Ann. Med. 41, 109–119. 10.1080/07853890802337045. 18720093

[13] Byrd AL, Deming C, Cassidy SKB, Harrison OJ, Ng WI, Conlan S, NISC Comparative Sequencing Program; Belkaid Y., Segre JA., Kong HH. (2017). Staphylococcus aureus and Staphylococcus epidermidis strain diversity underlying pediatric atopic dermatitis. Sci. Transl. Med. 9, eaal4651. 10.1126/scitranslmed.aal4651. 28679656 PMC5706545

[14] Williams MR, Cau L, Wang Y, Kaul D, Sanford JA, Zaramela LS, Khalil S, Butcher AM, Zengler K, Horswill AR, (2020). Interplay of Staphylococcal and Host Proteases Promotes Skin Barrier Disruption in Netherton Syndrome. Cell Rep. 30, 2923–2933.e7. 10.1016/j.celrep.2020.02.021. 32130897 PMC7183042

[15] Liu H, Archer NK, Dillen CA, Wang Y, Ashbaugh AG, Ortines RV, Kao T, Lee SK, Cai SS, Miller RJ, (2017). Staphylococcus aureus Epicutaneous Exposure Drives Skin Inflammation via IL-36-Mediated T Cell Responses. Cell Host Microbe 22, 653–666.e5. 10.1016/j.chom.2017.10.006. 29120743 PMC5774218

[16] Nakagawa S, Matsumoto M, Katayama Y, Oguma R, Wakabayashi S, Nygaard T, Saijo S, Inohara N, Otto M, Matsue H, (2017). Staphylococcus aureus Virulent PSMalpha Peptides Induce Keratinocyte Alarmin Release to Orchestrate IL-17-Dependent Skin Inflammation. Cell Host Microbe 22, 667–677.e5. 10.1016/j.chom.2017.10.008. 29120744 PMC5728420

[17] Cau L, Williams MR, Butcher AM, Nakatsuji T, Kavanaugh JS, Cheng JY, Shafiq F, Higbee K, Hata TR, Horswill AR, and Gallo RL (2021). Staphylococcus epidermidis protease EcpA can be a deleterious component of the skin microbiome in atopic dermatitis. J. Allergy Clin. Immunol. 147, 955–966.e16. 10.1016/j.jaci.2020.06.024. 32634452 PMC8058862

[18] Monk IR, and Foster TJ (2012). Genetic manipulation of Staphylococci-breaking through the barrier. Front. Cell. Infect. Microbiol. 2, 49. 10.3389/fcimb.2012.00049. 22919640 PMC3417578

[19] Olson ME, Todd DA, Schaeffer CR, Paharik AE, Van Dyke MJ, Büttner H, Dunman PM, Rohde H, Cech NB, Fey PD, and Horswill AR (2014). Staphylococcus epidermidis agr quorum-sensing system: signal identification, cross talk, and importance in colonization. J. Bacteriol. 196, 3482–3493. 10.1128/JB.01882-14. 25070736 PMC4187671

[20] Rebholz B, Haase I, Eckelt B, Paxian S, Flaig MJ, Ghoreschi K, Nedospasov SA, Mailhammer R, Debey-Pascher S, Schultze JL, (2007). Crosstalk between keratinocytes and adaptive immune cells in an IkappaBalpha protein-mediated inflammatory disease of the skin. Immunity 27, 296–307. 10.1016/j.immuni.2007.05.024. 17692539

[21] Piipponen M, Li D, and Landén NX (2020). The Immune Functions of Keratinocytes in Skin Wound Healing. Int. J. Mol. Sci. 21, 8790. 10.3390/ijms21228790. 33233704 PMC7699912

[22] Albanesi C, Madonna S, Gisondi P, and Girolomoni G (2018). The Interplay Between Keratinocytes and Immune Cells in the Pathogenesis of Psoriasis. Front. Immunol. 9, 1549. 10.3389/fimmu.2018.01549. 30034395 PMC6043636

[23] Nguyen TH, Cheung GYC, Rigby KM, Kamenyeva O, Kabat J, Sturdevant DE, Villaruz AE, Liu R, Piewngam P, Porter AR, (2021). Rapid pathogen-specific recruitment of immune effector cells in the skin by secreted toxins. Nat. Microbiol. 7, 62–72. 10.1038/s41564-021-01012-9. 34873293 PMC8732318

[24] Nizet V, Ohtake T, Lauth X, Trowbridge J, Rudisill J, Dorschner RA, Pestonjamasp V, Piraino J, Huttner K, and Gallo RL (2001). Innate antimicrobial peptide protects the skin from invasive bacterial infection. Nature 414, 454–457. 10.1038/35106587. 11719807

[25] Bose JL, Fey PD, and Bayles KW (2013). Genetic tools to enhance the study of gene function and regulation in Staphylococcus aureus. Appl. Environ. Microbiol. 79, 2218–2224. 10.1128/AEM.00136-13. 23354696 PMC3623228

[26] Robinson MD, McCarthy DJ, and Smyth GK (2010). edgeR: a Bioconductor package for differential expression analysis of digital gene expression data. Bioinformatics 26, 139–140. 10.1093/bioinformatics/btp616. 19910308 PMC2796818

[27] McCarthy DJ, Chen Y, and Smyth GK (2012). Differential expression analysis of multifactor RNA-Seq experiments with respect to biological variation. Nucleic Acids Res. 40, 4288–4297. 10.1093/nar/gks042. 22287627 PMC3378882

[28] Chen Y, Lun ATL, and Smyth GK (2016). From reads to genes to pathways: differential expression analysis of RNA-Seq experiments using Rsubread and the edgeR quasi-likelihood pipeline. F1000Res. 5, 1438. 10.12688/f1000research.8987.2. 27508061 PMC4934518

[29] Love MI, Huber W, and Anders S (2014). Moderated estimation of fold change and dispersion for RNA-seq data with DESeq2. Genome Biol. 15, 550. 10.1186/s13059-014-0550-8. 25516281 PMC4302049

[30] Dobin A, Davis CA, Schlesinger F, Drenkow J, Zaleski C, Jha S, Batut P, Chaisson M, and Gingeras TR (2013). STAR: ultrafast universal RNA-seq aligner. Bioinformatics 29, 15–21. 10.1093/bioinformatics/bts635. 23104886 PMC3530905

[31] Patrick GJ, Liu H, Alphonse MP, Dikeman DA, Youn C, Otterson JC, Wang Y, Ravipati A, Mazhar M, Denny G, (2021). Epicutaneous Staphylococcus aureus induces IL-36 to enhance IgE production and ensuing allergic disease. J. Clin. Invest. 131, e143334. 10.1172/JCI143334. 33645549 PMC7919715

[32] Ewels P, Magnusson M, Lundin S, and Käller M (2016). MultiQC: summarize analysis results for multiple tools and samples in a single report. Bioinformatics 32, 3047–3048. 10.1093/bioinformatics/btw354. 27312411 PMC5039924

[33] Team., R. (2015). RStudio: Integrated Development Environment for R. Boston, MA.

[34] Wu T, Liu W, Huang S, Chen J, He F, Wang H, Zheng X, Li Z, Zhang H, Zha Z, (2021). clusterProfiler 4.0: A universal enrichment tool for interpreting omics data. Innovation 12, 100141. 10.1016/j.xinn.2021.100141. PMC8454663 34557778

[35] Yu G, Wang LG, Han Y, and He QY (2012). clusterProfiler: an R package for comparing biological themes among gene clusters. OMICS 16, 284–287. 10.1089/omi.2011.0118. 22455463 PMC3339379

