# Supplementary material for: Staphylococcus epidermidis activates keratinocyte cytokine expression and promotes skin inflammation through the production of phenol-soluble modulins
Source: Cell Rep. Author manuscript; Available in PMC 2023 Oct 19. (PMC10586132; doi:10.1016/j.celrep.2023.113024)
Supplement: 1 [file NIHMS1933878-supplement-1.pdf]

**Cell Reports, Volume *42***

## Supplemental information

# *Staphylococcus epidermidis* activates keratinocyte cytokine expression and promotes skin inflammation through the production of phenol-soluble modulins

**Michael R. Williams, Michelle D. Bagood, Timothy J. Enroth, Zoie L. Bunch, Nina Jiang, Edward Liu, Samia Almoughrabie, Shadi Khalil, Fengwu Li, Samantha Brinton, Nadja B. Cech, Alexander R. Horswill, and Richard L. Gallo**

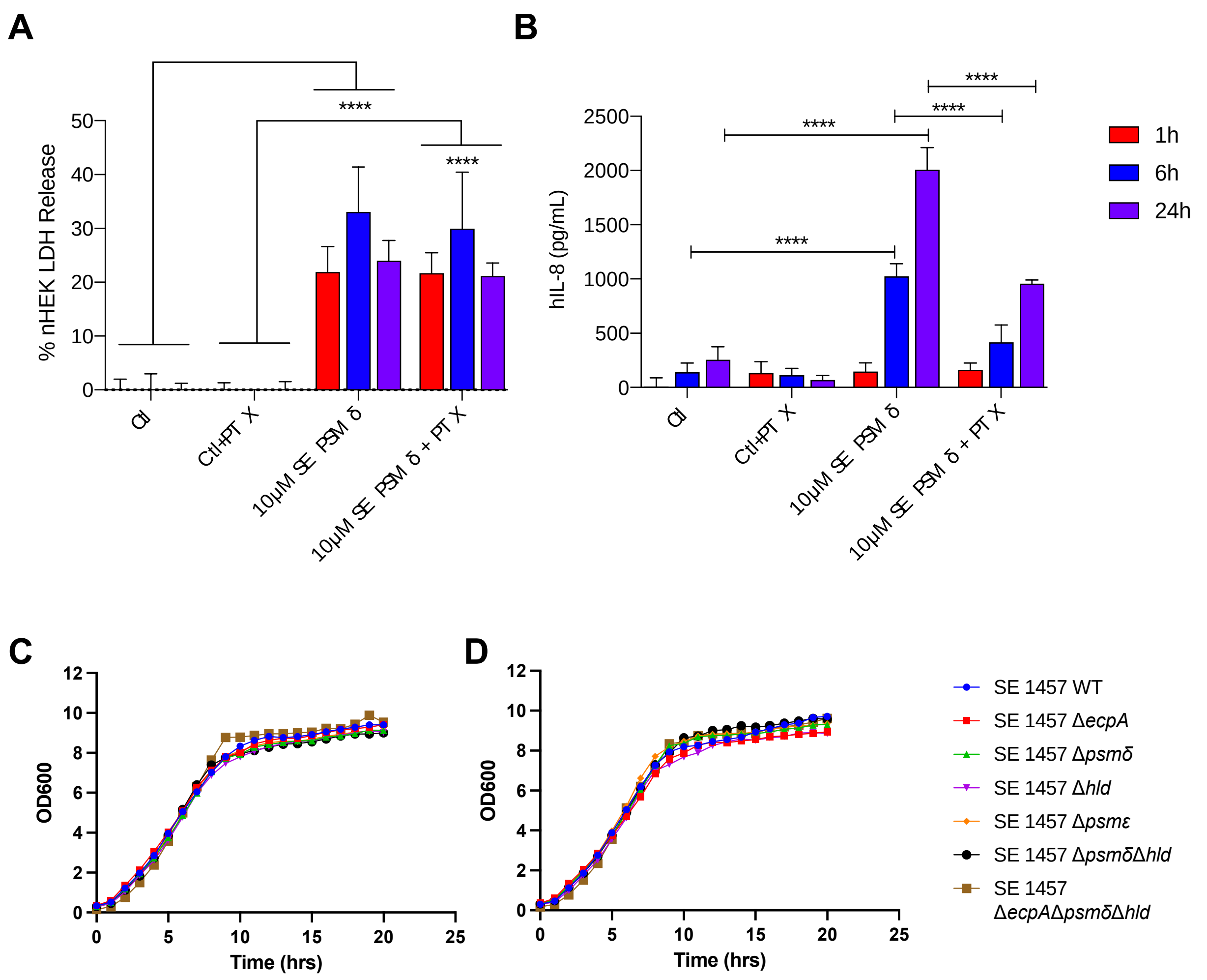


**Figure S1**. **Mechanism of synthetic PSM keratinocyte response and *S. epidermidis* mutant growth comparison, Related to Figure 1 and 2.** Primary human keratinocytes were treated for up to 24h with SE synthetic peptide PSMδ (10μM) with or without a 1μg/mL pertussis toxin (PTX) 30min pretreatment (n=4). **(A)** Assessment of LDH release and **(B)** hIL-8 ELISA protein levels from keratinocyte conditioned medium following treatment times. **(C)** Growth curve comparison of SE wild and mutant strains. **(D)** Repeat of growth curve comparison. Results are representative of at least two independent experiments. Mean ± SEM and a Two-way ANOVA analysis was used to determine statistical significance: p<0.05 *, p<0.01 **, p<0.001 ***, p<0.0001 ****.

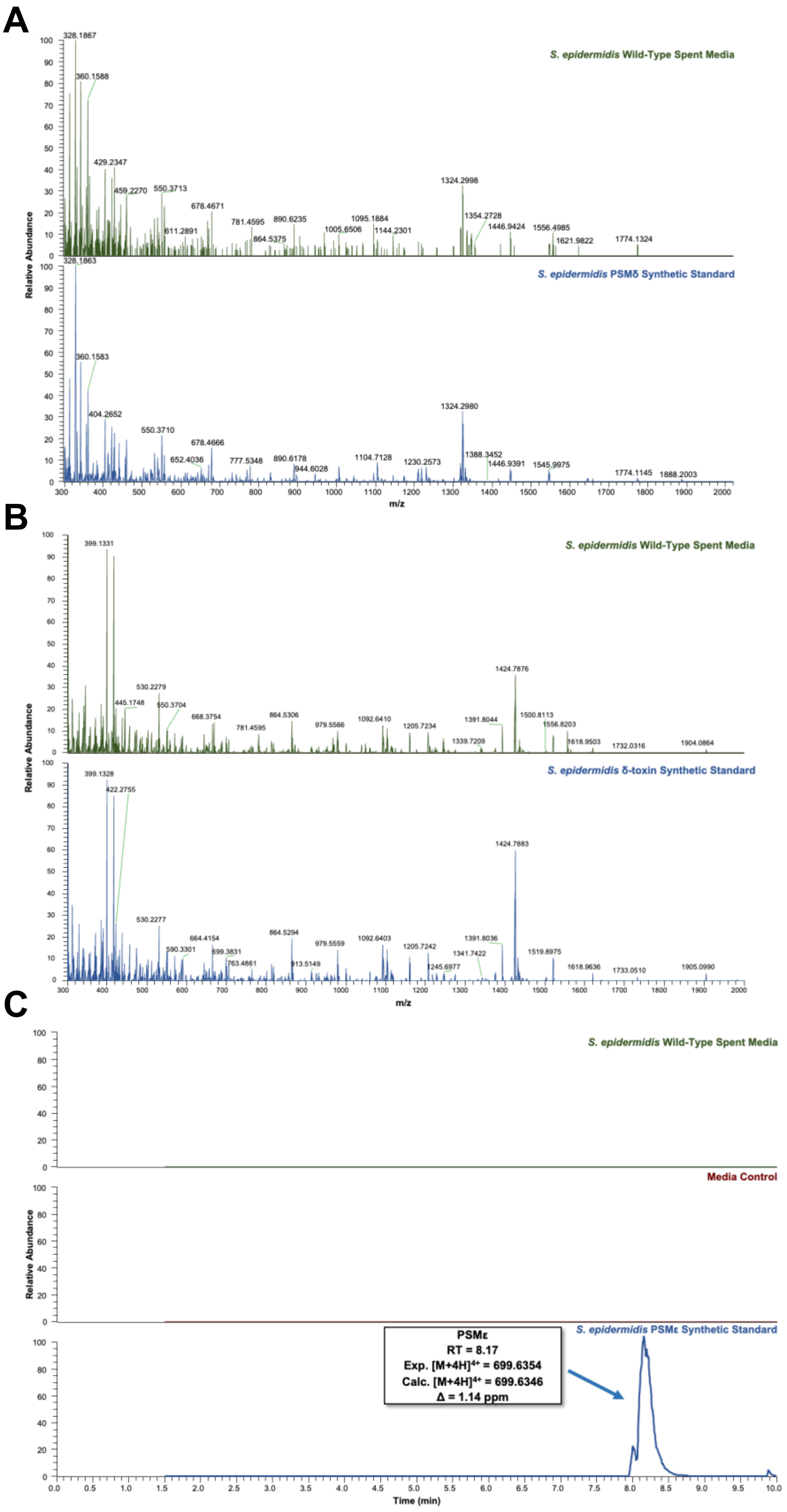
A
S. epidermidis Wild-Type Spent Media
S. epidermidis PSMδ Synthetic Standard
Relative Abundance
m/z
328.1867
360.1588
429.2347
459.2270
550.3713
678.4671
1324.2998
1354.2728
1446.9424
1556.4985
1621.9822
1774.1324
328.1863
360.1583
404.2652
550.3710
678.4666
1324.2980
1388.3452
1446.9391
1545.9975
1774.1145
1888.2003
B
S. epidermidis Wild-Type Spent Media
S. epidermidis δ-toxin Synthetic Standard
Relative Abundance
m/z
399.1331
1424.7876
399.1328
422.2755
1424.7883
C
S. epidermidis Wild-Type Spent Media
Media Control
S. epidermidis PSMε Synthetic Standard
Relative Abundance
Time (min)
PSMε
RT = 8.17
Exp. [M+4H]4+ = 699.6354
Calc. [M+4H]4+ = 699.6346
Δ = 1.14 ppm

**Figure S2**. **Mass spectrometric validation of *S. epidermidis* PSMδ, δ-toxin, and PSMε presence or absence in spent media, Related to Figure 2.** MS-MS analysis was used to validate the detection of PSMs in spent media. **(A)** Comparison of the fragmentation pattern for the *S. epidermidis* PSMδ in wild-type spent media (top) and for the synthetic standard (bottom) at a concentration of 50 µM. **(B)** Comparison of the fragmentation pattern for the *S. epidermidis* δ-toxin in wild-type spent media (top) and for the synthetic standard (bottom) at a concentration of 50 µM. **(C)** Selected ion chromatograms of the $[M+4H]^{4+}$ ion in *S. epidermidis* wild-type spent media (top), a negative media control (middle), and the synthetic standard (bottom). The mass error is calculated in ppm and the mass of the protonated peptide (Calc.) is shown in comparison to the experimental mass (Exp.). Chromatograms are normalized to a signal of 4.00 x $10^8$.

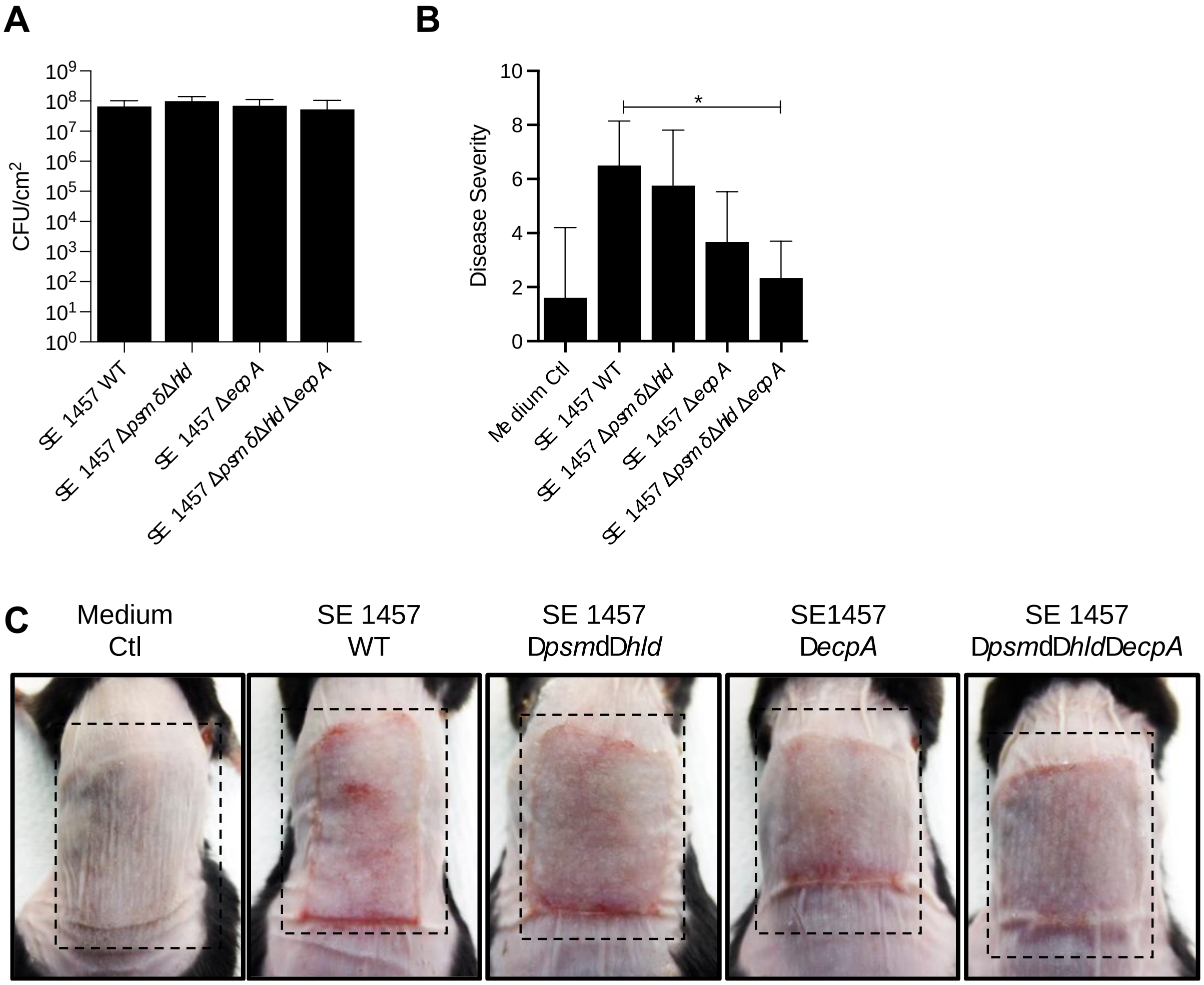


**Figure S3**. **Early live SE treatment of murine back skin, Related to Figure 4.** Murine dorsal skin treated with epicutaneous application of 1 x $10^7$ CFU/$cm^2$ of live SE strain 1457 wild-type or mutant strains SE Δ*psmδ*Δ*hld*, SEΔ*ecpA*, or SE Δ*psmδ*Δ*hld*Δ*ecpA* for 24 hrs (n=6 per group). **(A)** Assessment of CFU/$cm^2$ of bacteria from mice following 24 hrs treatment. **(B)** Assessment of disease severity on murine back skin. **(C)** Visual representation of topical back skin inflammation. Results are representative of at least two independent experiments. Mean ± SEM and a nonparametric unpaired Kruskal-Wallis analysis was used to determine statistical significance: $p<0.05$ *, $p<0.01$ **, $p<0.001$ ***, $p<0.0001$ ****.

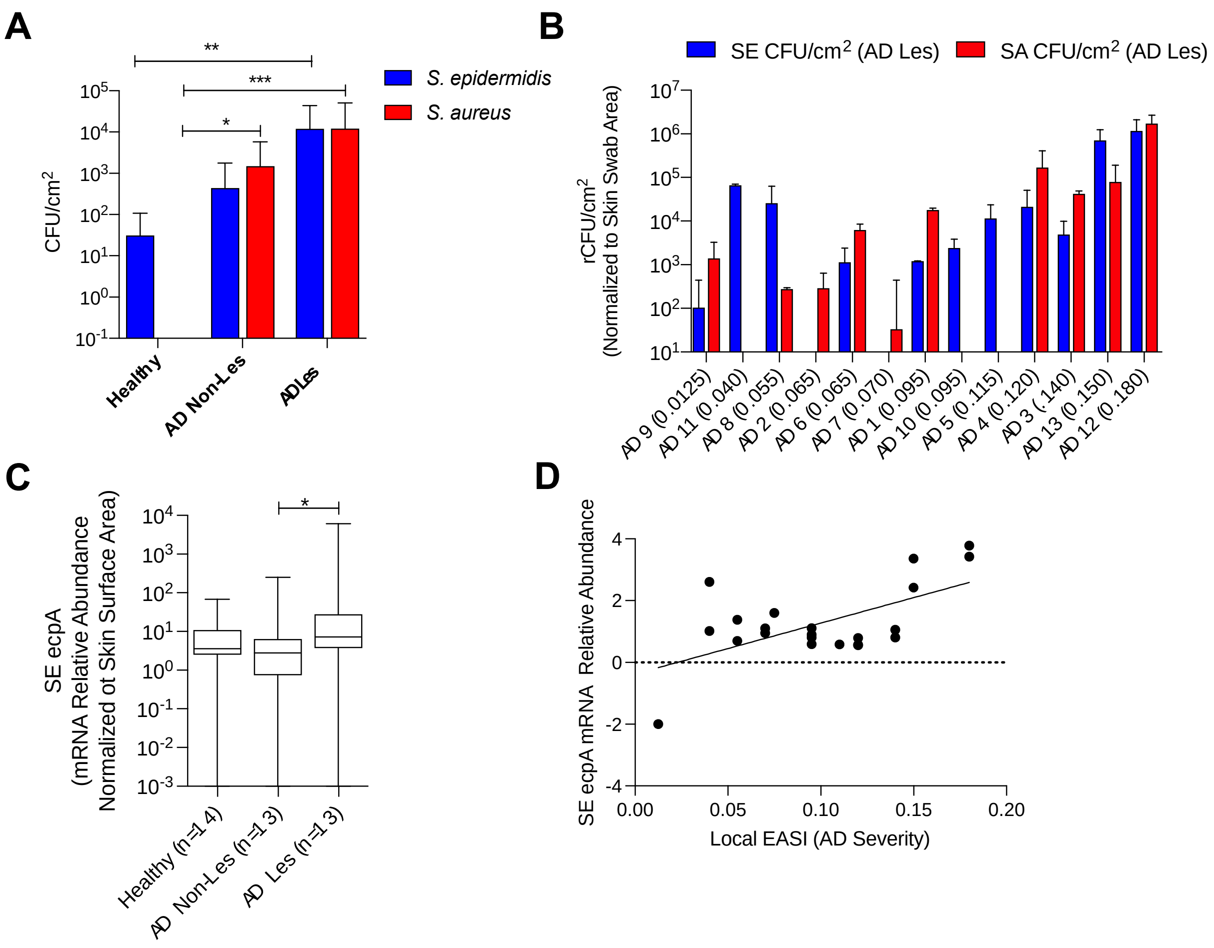


**Figure S4**. **SE absolute abundance compared to *S. aureus* from human skin swabs, Related to Figure 5.** qPCR analysis of SE and *S. aureus* gDNA absolute abundance (CFU/cm$^2$) levels from skin swabs of healthy (n=14), AD Non-Les (n=13) and AD Les (n=13). **(A)** Combined analysis of gDNA microbe levels by condition and **(B)** assessment of lesional atopic dermatitis (AD) skin swab levels by least to most severe local EASI disease severity scores. Mean ± SEM and a non-parametric unpaired Kruskal-Wallis analysis was used to determine statistical significance: p<0.05 *, p<0.01 **, p<0.001 ***, p<0.0001 ****.
